# Supplementary material for: Nuclear export receptor CRM1 recognizes diverse conformations in nuclear export signals
Source: eLife. 2017 Mar 10;6:e23961. doi: 10.7554/eLife.23961 (PMC5358978; doi:10.7554/eLife.23961)
Supplement: Figure 1—source data 1. — DOI: http://dx.doi.org/10.7554/eLife.23961.003 [file elife-23961-fig1-data1.docx]

**Figure 1 – source data 1.** Data collection and refinement statistics

| **Data collection** | | | | | |
| --- | --- | --- | --- | --- | --- |
| Crystal of CRM1*-Ran-RanBP1 bound to: | HDAC5^NES^ | Paxillin^NES^ | FMRP-1b^NES^ | FMRP^NES^ | SMAD4^NES^ |
| Space group | P4_3_2_1_2 | | | | |
| Cell dimensions a=b, c (Å) | 106.39, 304.72 | 106.98, 304.88 | 106.48, 304.3 | 106.49, 303.93 | 106.39, 304.22 |
| Resolution range (Å) | 50.00 – 2.14 (2.18 – 2.14) | 50.00 – 2.26 (2.30 – 2.26) | 50.00 – 2.35 (2.39 – 2.35) | 50.00 – 2.22 (2.26 – 2.22) | 50.00 – 2.24 (2.28 – 2.24) |
| Multiplicity | 6.5 (6.4) | 7.3 (7.0) | 8.6 (7.7) | 6.2 (6.3) | 6.0 (5.9) |
| Data completeness (%) | 98.8 (99.7) | 91.8 (92.7) | 100 (100) | 99.4 (100) | 99.8 (100) |
| *R*_merge_ /*R*_pim_ (%) | 6.5 (95.6) / 2.7 (40.2) | 8.1 (>100) / 3.1 (46.1) | 10.9 (>100) / 3.9 (57.1) | 7.7 (96.6) / 3.3 (40.8) | 7.1 (90.8) / 3.1 (40.6) |
| I/σ(I) | 26.4 (2.0) | 22.8 (1.7) | 19.0 (1.2) | 21.6 (2.1) | 22.9 (2.0) |
| CC_1/2_ (last resolution shell)^a^ | 0.679 | 0.621 | 0.444 | 0.669 | 0.662 |
| **Refinement statistics** | | | | | |
| Resolution range (Å) | 47.12 – 2.14 (2.20 – 2.14) | 47.3 – 2.26 (2.31 – 2.26) | 40.1 – 2.35 (2.41 – 2.35) | 47.6 – 2.22 (2.28 – 2.22) | 47.6 – 2.24 (2.30 – 2.24) |
| No. of reflections *R*_work_/R_free_ | 94942/2000 (6014/129) | 74850/2000 (4149/114) | 67933/2000  (1744/53) | 86190/1852 (6084/135) | 83497/2000 (4810/118) |
| Data completeness (%) | 98.1 (91.0) | 88.92 (72.0) | 91.59 (34.0) | 98.96 (95.0) | 98.41 (83.0) |
| Atoms (non-H protein/ligand and ions/H_2_O) | 11046/57/735 | 11061/51/495 | 10935/45/545 | 11101/51/574 | 11047/52/543 |
| *R*_work_/*R*_free_ (%) | 18.3/21.8 (25.4/32.6) | 18.2/21.8  (24.7/29.9) | 18.8/22.3 (26.5/29.9) | 18.0/21.4 (24.2/28.2) | 18.1/21.1  (23.4/34.8) |
| R.m.s.d. Bond length (Å)/angle (°) | 0.002/0.536 | 0.003/0.554 | 0.003/0.597 | 0.004/0.631 | 0.003/0.575 |
| Mean B-value (Å^2^)^b^  Protein  Ligands and ions/H_2_O  NES peptide/Φs  Groove lining residues | 37.5  34.9  74.2/64.6  54.4 | 44.1  40.3  76.9/75.3  54.7 | 42.5  37.3  89.6/87.9  59.7 | 42.1  39.6  88.4/79.2  50.6 | 40.9  38.7  82.2/81.7  51.5 |
| Ramachandran plot  favored /disallowed (%)^c^ (chain and residue#) | 97.84/0.07  (B169) | 97.30/0.08  (C205, C306) | 96.75/0.15  (C134,C687) | 97.74/0.08  (C978) | 97.67/0.00 |
| ML coordinate error | 0.23 | 0.26 | 0.22 | 0.23 | 0.19 |
| Missing residues  Chain A: Ran  Chain B: RanBP1  Chain C: CRM1  Chain D: NES peptide | A: 1-8; B: 62-63, 70-77, 201; C: 440-456, 1054-1058 | A: 1-8, 188-192; B: 62-63, 69-77, 199-201; C: 442-456, 1054-1058; D: 264, 279-281 | A: 1-8, 187-189; B: 62, 69-77, 200-201; C: 440-460, 1054-1058; D: 1-10 | A: 1-8, 188, 189; B: 62, 70-77, 201; C: 440-460, 1054-1058; D: 423-426 | A: 1-8; B: 62, 70-77, 201; C: 441-460, 1054-1058; D: 133-140 |
| PDB code | 5UWI | 5UWH | 5UWO | 5UWJ | 5UWU |

Data for the outermost shell are given in parentheses.

^a^ Karplus PA & Diederichs K (2012) Linking crystallographic model and data quality. Science 336(6084):1030-1033.

^b^ B-factors for the entire NES peptide, B-factors for only Φ residues of the NES peptides (as indicated in the figures) and B-factors for the 29 CRM1 residues that line the NES-binding groove are also reported.

^c^ As defined by MolProbity in PHENIX.
