# Supplementary material for: Nuclear export receptor CRM1 recognizes diverse conformations in nuclear export signals
Source: eLife. 2017 Mar 10;6:e23961. doi: 10.7554/eLife.23961 (PMC5358978; doi:10.7554/eLife.23961)
Supplement: Figure 1—source data 2. — DOI: http://dx.doi.org/10.7554/eLife.23961.004 [file elife-23961-fig1-data2.docx]

**Figure 1 – source data 2.** Data collection and refinement statistics (cont.)

| **Data collection** | | | | |
| --- | --- | --- | --- | --- |
| Crystal of CRM1*-Ran-RanBP1 bound to: | mDia2^NES^ | CDC7^NES^ | CDC7^NES^ ext | X11L2^NES^ |
| Space group | P4_3_2_1_2 | | | |
| Cell dimensions a=b, c (Å) | 106.18, 303.32 | 106.39, 304.57 | 106.09, 304.55 | 106.65, 304.63 |
| Resolution range (Å) | 50.00 – 2.05 (2.09 – 2.05) | 50.00 – 2.28 (2.32 – 2.28) | 50.00 – 2.24 (2.28 – 2.24) | 50.00 – 2.40 (2.44 – 2.40) |
| Multiplicity | 6.4 (6.5) | 5.7 (5.7) | 12.6 (12.0) | 9.4 (7.3) |
| Data completeness (%) | 99.9 (100) | 99.0 (98.5) | 99.4 (99.0) | 99.9 (100) |
| *R*_merge_ /*R*_pim_ (%) | 7.9 (95.7) / 3.4 (40.9) | 7.4 (>100) / 3.8 (54.2) | 11.4 (>100) / 3.3 (50.3) | 9.4 (>100) / 4.1 (60.5) |
| I/σ(I) | 23.2 (1.9) | 20.5 (1.6) | 23.8 (1.5) | 24.4 (1.7) |
| CC_1/2_ (last resolution shell)^a^ | 0.668 | 0.578 | 0.384 | 0.672 |
| **Refinement statistics** | | | | |
| Resolution range (Å) | 36.45 – 2.05 (2.11 – 2.05) | 40.3 – 2.28 (2.34 – 2.28) | 47.45 – 2.24 (2.30 – 2.24) | 47.7 – 2.40 (2.46 – 2.40) |
| No. of reflections *R*_work_/R_free_ | 105803/2000 (5065/98) | 77105/1999 (3800/101) | 79116/2000 (3436/89) | 67967/2003 (3397/103) |
| Data completeness (%) | 97.23 (67.0) | 95.39 (69.0) | 93.57 (60.0) | 97.57 (71.0) |
| Atoms (non-H protein/ligand and ions//H_2_O) | 10997/51/889 | 11066/57/516 | 10960/52/522 | 11004/51/321 |
| *R*_work_/*R*_free_ (%) | 17.8/21.3 (24.8/32.0) | 18.5/22.5 (24.4/31.2) | 18.7/22.2 (25.0/27.4) | 18.8/22.2 (25.1/28.7) |
| R.m.s.d.  Bond length (Å)/angle (°) | 0.003/0.547 | 0.002/0.535 | 0.003/0.527 | 0.003/0.479 |
| Mean B-value (Å^2^)^b^  Protein  Ligands and ions/H_2_O  NES peptide/Φs  Groove lining residues | 37.3  36.9  85.8/88.7  59.4 | 43.7  39.5  86.7/79.6  63.5 | 42.4  36.44  96.6/93.0  68.9 | 48.0  44.9  65.4/60.7  52.5 |
| Ramachandran plot  favored /disallowed (%)^c^  (chain and residue#) | 98.05/0.08  (A9) | 97.00/0.00 | 97.74/0.00 | 97.83/0.07  (C266) |
| ML coordinate error | 0.21 | 0.26 | 0.25 | 0.26 |
| Missing residues  Chain A: Ran  Chain B: RanBP1  Chain C: CRM1  Chain D: NES peptide | A: 1-7; B: 62-63, 69-77, 201; C: 440-460, 1054-1058; D: 1179-1182 | A: 1-8, 188, 189; B: 62, 70-79, 201; C: 442-456, 1054-1058; D: 456, 470-473 | A: 1-8, 188-191; B: 62-63, 69-78, 201; C: 440-456, 1053-1058; D: 456, 469-478 | A: 1-8, 188, 189; B: 62, 69-77; C: 440-460, 1054-1058; D: 55 |
| PDB code | 5UWP | 5UWQ | 5UWR | 5UWS |

Data for the outermost shell are given in parentheses.

^a^ Karplus PA & Diederichs K (2012) Linking crystallographic model and data quality. Science 336(6084):1030-1033.

^b^ B-factors for the entire NES peptide, B-factors for only Φ residues of the NES peptides (as indicated in the figures) and B-factors for the 29 CRM1 residues that line the NES-binding groove are also reported.

^c^ As defined by the validation suite MolProbity in PHENIX.
