# Supplementary material for: Nuclear export receptor CRM1 recognizes diverse conformations in nuclear export signals
Source: eLife. 2017 Mar 10;6:e23961. doi: 10.7554/eLife.23961 (PMC5358978; doi:10.7554/eLife.23961)
Supplement: Figure 1—source data 3. — DOI: http://dx.doi.org/10.7554/eLife.23961.005 [file elife-23961-fig1-data3.docx]

**Figure 1 – source data 3.** Crystallization conditions of CRM1-NES complexes

|  | Peptide sequence | Crystallization condition |
| --- | --- | --- |
| HDAC5 | GGSY-^1082^EAETVSAMALLSVG^1095^ | 17% PEG3350, 100mM Bis-Tris pH 6.4, 200mM NH_4_NO_3_, 10mM Spermine HCl, 16mM HCl |
| Paxillin (isoform α) | GGSY-^264^RELDELMASLSDFKFMAQ^281^ | 16% PEG3350, 100mM Bis-Tris pH 6.4, 200mM NH_4_NO_3_, 20mM HCl |
| FMRP-1b | GGS-YLKEVDQLRALERLQID | 16% PEG3350, 100mM Bis-Tris pH 6.4, 200mM NH_4_NO_3_, 12mM HCl |
| FMRP | ^423^YLKEVDQLRLERLQI^437^ | 17% PEG3350, 100mM Bis-Tris pH 6.4, 200mM NH_4_NO_3_, 10mM Spermine HCl, 4mM HCl |
| SMAD4 | GGS-^133^YERVVSPGIDLSGLTLQ^149^ | 16% PEG3350, 100mM Bis-Tris pH 6.4, 200mM NH_4_NO_3_, 16mM HCl |
| mDia2 | GGSY-^1179^SVPEVEALLARLRAL^1193^ | 16% PEG3350, 100mM Bis-Tris pH 6.4, 200mM NH_4_NO_3_, 8mM HCl |
| CDC7 | GGSY-^456^QDLRKLCERLRGMDSSTP^473^ | 16% PEG3350, 100mM Bis-Tris pH 6.4, 200mM NH_4_NO_3_, 8mM HCl |
| CDC7-ext | GGSY-^456^QDLRKLCERLRGMDSSTPKLTSD^478^ | 17% PEG3350, 100mM Bis-Tris pH 6.4, 200mM NH_4_NO_3_, 10mM Spermine HCl |
| X11L2 | GGSY-^55^SSLQELVQQFEALPGDLV^72^ | 17% PEG3350, 100mM Bis-Tris pH 6.4, 200mM NH_4_NO_3_, 10mM Spermine HCl |
