# Supplementary material for: Nuclear export receptor CRM1 recognizes diverse conformations in nuclear export signals
Source: eLife. 2017 Mar 10;6:e23961. doi: 10.7554/eLife.23961 (PMC5358978; doi:10.7554/eLife.23961)
Supplement: Figure 4—source data 1. — DOI: http://dx.doi.org/10.7554/eLife.23961.019 [file elife-23961-fig4-data1.docx]

**Figure 4 – source data 1.** Data collection and refinement statistics and crystallization conditions.

| **Data collection** | | |
| --- | --- | --- |
| Crystal of CRM1* K579A -Ran-RanBP1 bound to: | Hxk2^pep^ | DEAF1^pep^ |
| Space group | P4_3_2_1_2 | |
| Cell dimensions a=b, c (Å) | 106.71, 304.79 | 106.04, 304.21 |
| Resolution range (Å) | 50.00 – 2.34 (2.38 – 2.34) | 50.00 – 2.15 (2.19 – 2.15) |
| Multiplicity | 7.2 (7.2) | 13.7 (13.8) |
| Data completeness (%) | 100 (100) | 100 (100) |
| *R*_merge_ /*R*_pim_ (%) | 10.5 (>100) / 4.2 (51.9) | 9.0 (>100) / 2.5 (54.3) |
| I/σ(I) | 18.9 (1.4) | 32.2 (1.5) |
| CC_1/2_ (last resolution shell)^a^ | 0.511 | 0.526 |
| **Refinement statistics** | | |
| Resolution range (Å) | 47.7 – 2.34 (2.40 – 2.34) | 40.2 – 2.15 (2.21 – 2.15) |
| No. of reflections *R*_work_/R_free_ | 68199/1999 (1714/52) | 87729/2000 (1957/45) |
| Data completeness (%) | 90.89 (34.0) | 92.34 (30.0) |
| Atoms (protein/ions/water) | 10973/57/471 | 11042/51/720 |
| *R*_work_/*R*_free_ (%) | 18.7/22.7 (24.8/24.8) | 18.6/22.6 (25.9/35.2) |
| R.m.s.d.  Bond length (Å)/ angle (°) | 0.003/0.468 | 0.002/0.515 |
| Mean B-value (Å^2^)^b^  Protein  Ligands and ions/water  NES peptide/Φs  Groove lining residues | 45.2  40.8  107.8/97.0  63.1 | 35.8  31.7  89.5/83.2  55.5 |
| Ramachandran plot  favored /disallowed (%)^c^  (chain and residue#) | 97.76/0.00 | 97.01/0.07  (C205) |
| ML coordinate error | 0.27 | 0.21 |
| Missing residues  Chain A: Ran  Chain B: RanBP1  Chain C: CRM1  Chain D: NES peptide | A: 1-8; B: 62-64, 69-77, 201; C: 441-456, 1054-1058; D: 18,36 | A: 1-8,188, 189; B: 62-63, 69-78; C: 445-455, 1053-1058; D: 452, 453, 465-469 |
| PDB code | 5UWT | 5UWW |
| Peptide used | GGSY-^18^DVPKELMQQIENFEKIFTV^36^ | GGS-^452^SWLYLEEMVNSLLNTAQQ^469^ |
| Crystallization condition | 17% PEG3350, 100mM Bis-Tris pH 6.4, 200mM NH_4_NO_3_, 20mM HCl | 16% PEG3350, 100mM Bis-Tris pH 6.4, 200mM NH_4_NO_3_, 16mM HCl |

Data for the outermost shell are given in parentheses.

^a^ Karplus PA & Diederichs K (2012) Linking crystallographic model and data quality. Science 336(6084):1030-1033.

^b^ B-factors for the entire NES peptide, B-factors for only Φ residues of the NES peptides (as indicated in the figures) and B-factors for the 29 CRM1 residues that line the NES-binding groove are also reported.

^c^ As defined by the validation suite MolProbity in PHENIX.
